# Animal Management at the Ancient Metropolis of Teotihuacan, Mexico: Stable Isotope Analysis of Leporid (Cottontail and Jackrabbit) Bone Mineral

**DOI:** 10.1371/journal.pone.0159982

**Published:** 2016-08-17

**Authors:** Andrew D. Somerville, Nawa Sugiyama, Linda R. Manzanilla, Margaret J. Schoeninger

**Affiliations:** 1 Department of Anthropology, University of California San Diego, 9500 Gilman Drive, La Jolla, CA, 92093-0532, United States of America; 2 Department of Sociology and Anthropology, George Mason University, SOAN, MSN: 3G5, Fairfax, VA, 22030, United States of America; 3 Instituto de Investigaciones Antropológicas, Universidad Nacional Autónoma de México, Ciudad Universitaria, Mexico City, 04510, Distrito Federal, Mexico; University of Florence, ITALY

## Abstract

Human-animal interactions have played crucial roles in the development of complex societies across the globe. This study examines the human-leporid (cottontail and jackrabbit) relationship at the pre-Hispanic (AD 1–550) city of Teotihuacan in the Basin of Mexico and tests the hypothesis that leporids were managed or bred for food and secondary products within the urban core. We use stable isotope analysis (*δ*^13^C_apatite_ and *δ*^18^O_apatite_) of 134 leporid specimens from five archaeological contexts within the city and 13 modern specimens from across central Mexico to quantify aspects of leporid diet and ecology. The results demonstrate that leporids from Oztoyahualco, a residential complex associated with a unique rabbit sculpture and archaeological traces of animal butchering, exhibit the highest *δ*^13^C_apatite_ values of the sample. These results imply greater consumption of human-cultivated foods, such as maize (*Zea mays*), by cottontails and jackrabbits at this complex and suggest practices of human provisioning. A lack of significant differences in *δ*^18^O_apatite_ values between ancient and modern leporids and between Oztoyahualco and other locations within Teotihuacan indicates generally similar relative humidity from sampled contexts. Results of this study support the notion that residents provisioned, managed, or bred leporids during the height of the city, and provide new evidence for mammalian animal husbandry in the ancient New World.

## Introduction

Mutualistic relationships between humans and terrestrial herbivores have played critical roles in the history and development of complex societies. The coevolution of Eurasian and African societies with ungulates, such as cows (*Bos* spp.), goats (*Ovid*. spp.) and sheep (*Caprid* spp.) has resulted in their domestication for transportation, meat, and secondary products, and in drastic transformations to natural landscapes [1–4]. Fewer large mammals suitable for domestication were available in Pre-Columbian North and Central America [5], and their absence has led some to explore constraints on the growth of New World cities including the limits of long-distance trade networks [6] and the availability of high-quality protein [7–9].

**Fig 1:**
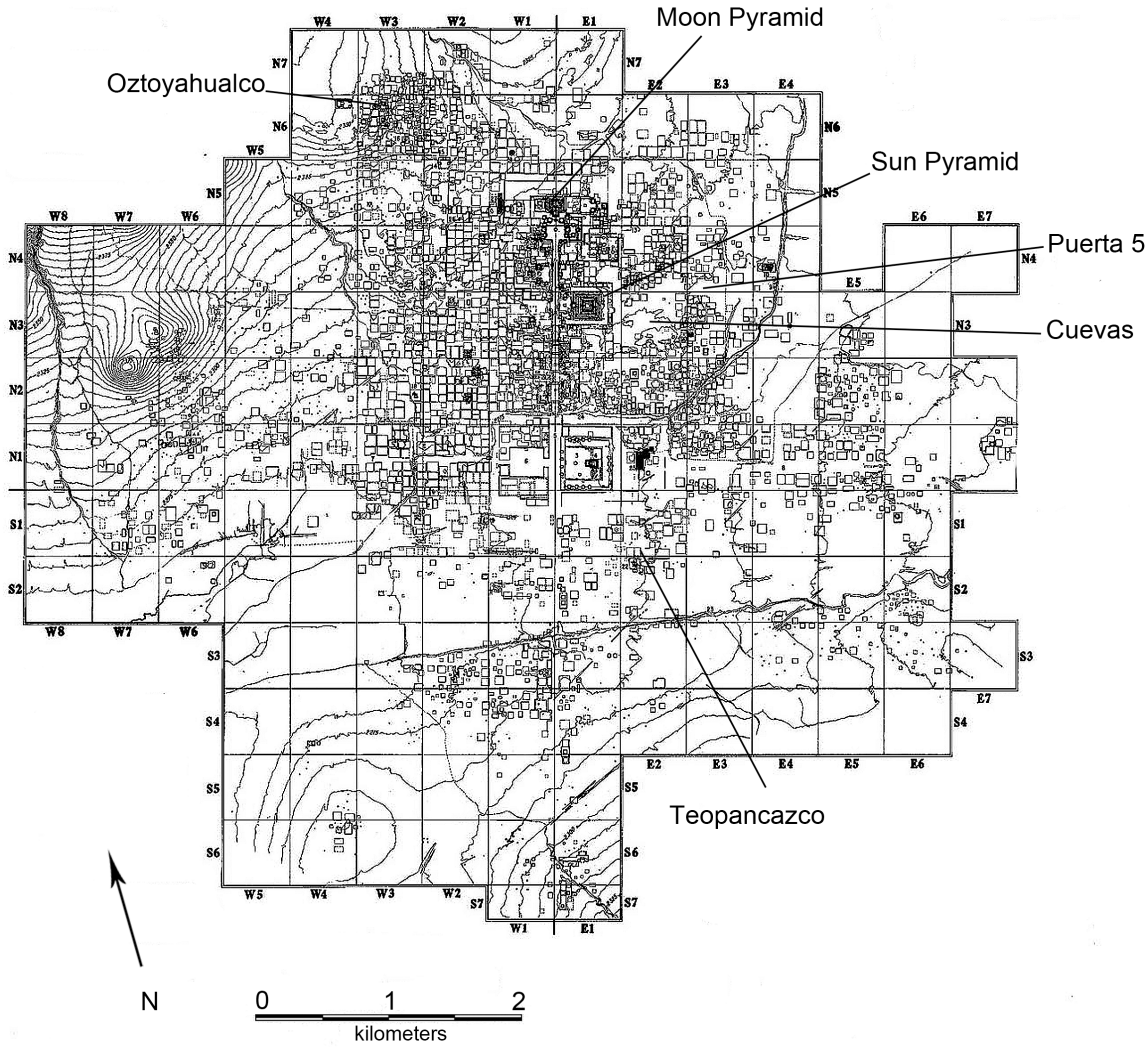
Map of Teotihuacan highlighting site locations mentioned in the text (After Millon 1973).

Located in the northeast of the Basin of Mexico, the UNESCO World Heritage site of Teotihuacan (AD 1–550; Fig 1) covered 20 km^2^ and possessed a population of approximately 100,000 residents, making it the largest urban center of its time [10, 11]. The site, which has been the subject of numerous archaeological excavations over the past century, presents an opportunity to study human-animal interactions at a major pre-Hispanic city of Mexico. Faunal analyses of excavated materials from Teotihuacan demonstrate that wild leporids (cottontails and jackrabbits) were among the most frequently represented mammals, suggesting their importance to the nutritional and economic history of the city [12, 13]. By using stable isotope analysis of leporid bones from multiple locations across the urban core, this study produces quantitative data on the diet and ecology of these small mammals in an effort to contribute to our growing knowledge of the importance of human-animal interactions at an ancient urban center.

**Fig 2:**
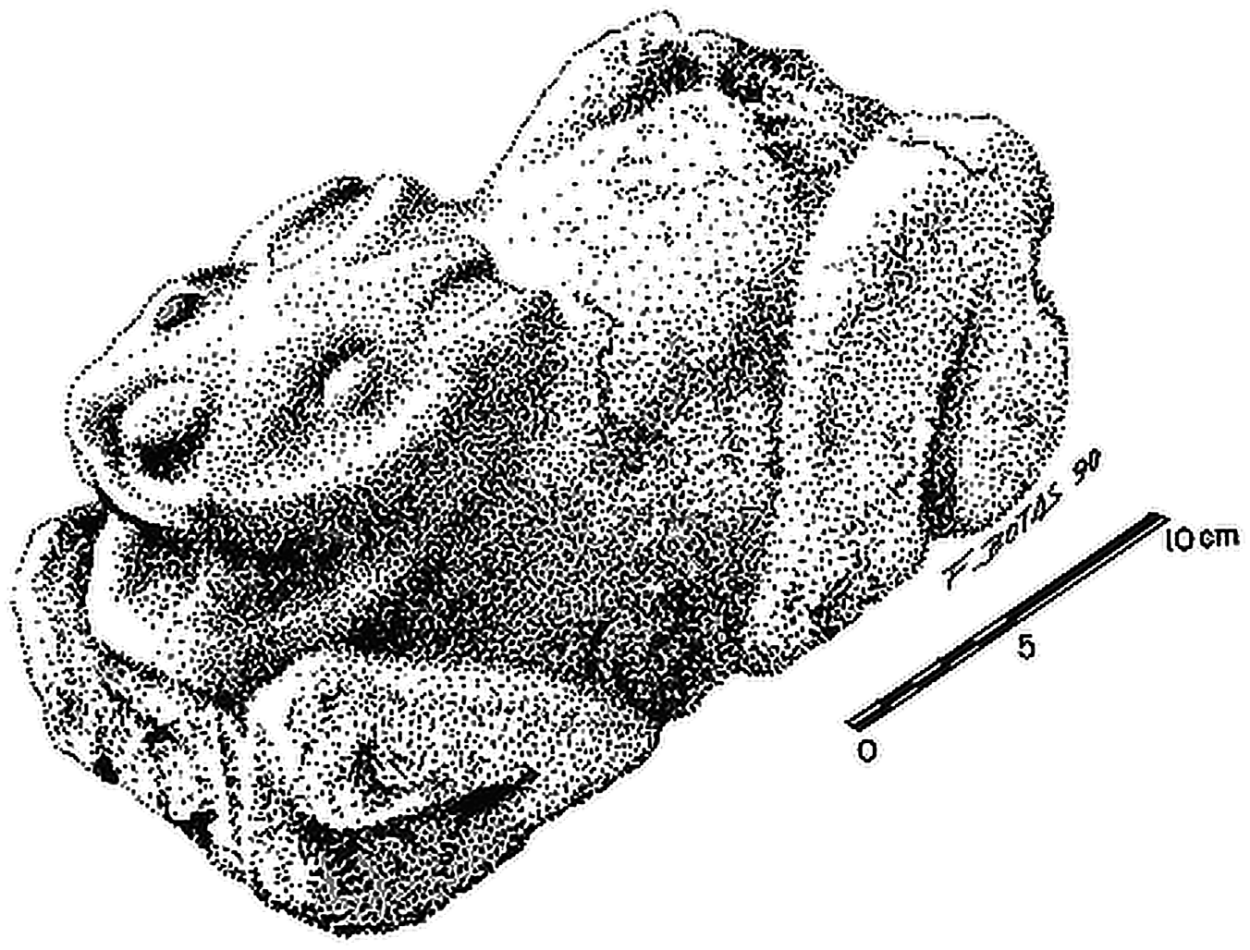
Illustration of stone rabbit sculpture from the Oztoyahualco 15B apartment compound. (Manzanilla ed.1993; drawing by Fernando Botas).

The high population at Teotihuacan and the overhunting of large mammals from the local landscape may have favored the exploitation of smaller game during the period peak occupation in the valley [14]. Indeed, archaeological evidence indicates that deer were more commonly consumed prior to the rise of the city, but that they were less commonly encountered during the Teotihuacan era, replaced by a broader spectrum of smaller animals [14]. Previous excavations within the urban core hint that cottontails and jackrabbits may have been managed and bred at select locations during the Classic period (AD 200–600) [15–17]. In particular, the Xolalpan phase (AD 350–550) apartment compound of Oztoyahualco 15B, located in the northwest portion of the site (15B:N6W3), contained a relatively large assemblage of cottontail and jackrabbit remains, accounting for approximately half of all identified fauna: 48% using the minimum number of individuals (MNI) [17]. Several rooms with high soil phosphate levels in the floor suggest the presence of disintegrated fecal matter or blood from butchering [18], and a unique stone sculpture of a rabbit was found within one of the three public courtyards of the complex (Fig 2). Rooms 9 and 10 of Oztoyahualco 15B, in particular, display the most evidence of leporid management. Both rooms exhibit high soil phosphate levels [18], some of the highest concentrations of obsidian blades within the complex [19], and neither appears to have been used for habitation. Room 9 may have been a location for butchering rabbits as multiple foot and limb bones were discovered as well as 58 obsidian blades and a half-sphere of decorated dolomite groundstone, perhaps serving as an “anvil” for cutting hides [19]. Excavations of Room 10 revealed obsidian blades in all stages of manufacturing and 50% of all the leporid remains from the compound, suggesting its association with leporid butchering [19]. In its southwest corner, Room 10 connected to Room 30, a small rectangular unit (1.20 x 1.25 m) with low volcanic stone walls (Room 30), suggestive of a pen for domestic animal management [17].

To investigate long-term dynamics of human-leporid ecology at Teotihuacan, this study uses stable carbon and oxygen isotope analysis of leporid bone mineral (*δ*^13^C_apatite_ and *δ*^18^O_apatite_) from multiple contexts within the city, and from a sample of modern specimens from central Mexico. Both spatial and temporal scales are considered, attempting to situate the dynamics of human-leporid interactions within their historical and ecological contexts. More specifically, stable isotope data are used to test the hypothesis that leporids from Oztoyahualco 15B were directly managed/bred by humans during the Xolalpan period (AD 350–550), which resulted in their greater consumption of human-cultivated foods than leporids from other contexts and times.

## Isotope Ecology in Highland Mexico

Carbon isotope ratios of well-preserved bone are principally determined by the photosynthetic pathway of consumed plants, which can be C3, C4, or Crassulacean acid metabolism (CAM) [20, 21]. Most plants utilize the C3 photosynthetic pathway, including all trees, forbs, and shrubs. These plants exhibit a global *δ*^13^C average of approximately -27‰ [22–24]. C4 plants primarily include dry adapted grasses, and exhibit much higher (less negative) *δ*^13^C values than C3 plants, with a global average of approximately -12.5‰ [23]. CAM plants include cacti and succulents and exhibit values similar to C4 plants within highland Mexico [23, 25]. Notably, many of the most important plants cultivated by humans in pre-Hispanic Mexico [26], such as maize (*Zea mays*), amaranth (*Amaranthus* spp.), nopal cactus (*Opuntia* spp.) and maguey (*Agave* spp.), were either C4 or CAM and are characterized by relatively high *δ*^13^C [23, 25]. Carbon isotope ratios from bone mineral apatite (*δ*^13^C_apatite_) reflect a mixture of the *δ*^13^C values from the total diet [27, 28]. Based on results of controlled feeding studies of modern rodents, *δ*^13^C_apatite_ is assumed to be approximately 9.7‰ higher than dietary *δ*^13^C [29]. Leporid *δ*^13^C_apatite_ values are thus considered representative the relative proportion of dietary C3 and C4/CAM plants consumed over the entire life history of selected specimens, which is commonly between 1–3 years [30, 31].

Stable carbon isotope values, then, may be used to represent the degree of integration of leporids into human-dominated social-environmental systems under the assumption that higher bone *δ*^13^C values represent greater consumption of human-cultivated food staples [32–34]. At other pre-Columbian archaeological sites in North America, previous studies have demonstrated that captive parrots [33], turkeys [32], and dogs [34] displayed high *δ*^13^C values, indicative of maize consumption. In fact, recent stable isotope analyses on carnivore remains from Teotihuacan itself found that captive carnivores displayed higher *δ*^13^C_apatite_ values than captive equivalents by about 6‰ [35].

Because agricultural landscapes around Teotihuacan were likely largely dedicated to producing C4/CAM plants, and because the modern reference sample was acquired through the hunting of wild specimens by the U.S. Geological Survey in more forested environments during a period of lower human population, we hypothesize that stable carbon isotope values of ancient specimens will exhibit higher values than modern specimens. Focusing explicitly on the question of leporid management within the city, we hypothesize that Oztoyahualco specimens will exhibit higher *δ*^13^C_apatite_ values than leporids from all other contexts due to their direct provisioning of human-cultivated foodstuffs.

A potential confound to stable carbon isotope analysis is climatic variation through time, which may influence the local proportion of C3 vs C4/CAM plants in the region. Indeed, paleosol studies from McClung de Tapia et al. [36] provide some evidence for environmental variability during the occupation of Teotihuacan. C4/CAM plants are dry-adapted species and flourish under conditions of increasing temperature and decreasing moisture [37–39]. Extended periods of reduced rainfall, humidity, and increased sunlight might promote the spread of these plants, serving to raise baseline *δ*^13^C values of the local environment and hence leporid values. This factor may partially be controlled for through analysis of stable oxygen isotope analysis.

Stable oxygen isotope ratios from bone mineral bioapatite (*δ*^18^O_apatite_), are primarily influenced by *δ*^18^O values of consumed water sources [40, 41]. Non-obligate drinking organisms, such as the Leporidae, obtain water primarily through consumed vegetal material and exhibit *δ*^18^O_apatite_ values that correlate negatively with relative humidity (RH) [42–44], as leaf water is sensitive to evaporative enrichment of ^18^O isotopes [45–47]. ^16^O is more readily lost through evaporation than ^18^O, leading to higher ^18^O/^16^O ratios in leaf water during periods of lower humidity. Experimental studies demonstrate that both C3 and C4 plants experience rising *δ*^18^O of leaf water and cellulose in response to decreasing relative humidity [48], and animals that acquire water primarily through plant material incorporate such patterns in skeletal tissue [42–44]. Previous research on a sample of European and African leporids confirms the significant negative relationship between enamel *δ*^18^O and local relative humidity [44]. Leporids living in Teotihuacan during times of greater humidity are thus expected to exhibit lower *δ*^18^O_apatite_ values than leporids living under more xeric conditions. This study made no assumptions regarding *δ*^18^O_apatite_ values and leporid breeding at Oztoyahualco, but used *δ*^18^O_apatite_ values as a rough control for the influence of climatic variation and its potential influence on the prevalence of C3 and C4/CAM plants in the region.

## Materials and Methods

### Leporidae

Jackrabbits and cottontails belong to the family Leporidae of the order Lagomorpha. Species of both genera were among the most commonly-consumed mammals by indigenous peoples of pre-Columbian North America. Both are attracted to human agricultural landscapes, and the raiding of cultivated fields and gardens of maize, beans, squash, and nopal cactus fruit may have been a significant component of the diet for leporids living near ancient urban environments. Like other agricultural societies of pre-Hispanic North America, we assume that residents of Teotihuacan acquired a large percentage of rabbits through “garden hunting”, a practice of opportunistic hunting for the purpose of pest elimination that would have also supplied a significant source of protein [49–51]. For hunting expeditions outside of local agricultural landscapes, research from the North American Southwest suggests that jackrabbits were more likely to be acquired through communal hunting drives while cottontails were more likely to be acquired by individual hunters [52].

Generally, wild *Lepus* species prefer open and desert habitats [53] while *Sylvilagus* species prefer habitats with more structure, including shrublands, shrub-woodlands, and areas with dense understory cover [54]. The genera have similar generalized foraging practices. As mixed feeders, both consume a variety of grasses, forbs, shrubs, and succulents, but the proportions of these plants in the diet vary by the time of year with the availability of seasonally green flora [55–60]. Both genera occupy relatively small home ranges, often remaining within 1 km^2^ for their entire lives. Jackrabbits typically inhabit areas between 20–140 hectares, grouping towards the smaller end of this range, while cottontails often inhabit ranges under 5 ha and rarely larger than 10 ha [58, 61, 62]. Jackrabbits and cottontails provide similar resources (e.g. meat, hide, fur, bones), have similar food requirements, and both may have been subject to human management or breeding. Nevertheless, due to their smaller size, shorter gestation period, and milder temperaments, it is possible that *Sylvilagus* would have been more amenable to reproduction in small enclosures than *Lepus* and an overall better candidate for active management and thus some consideration of differences between the genera are considered in our discussion.

### Leporid Bone Specimens

The sample included 134 leporid bones from multiple locations within the city of Teotihuacan and 13 modern specimens from the Mexican states of Puebla, Queretaro, and Distrito Federal. Stratigraphic association and radiocarbon dating demonstrated that leporids from archaeological contexts represent a temporal transect of approximately 1350 years that spans the growth and decline of the city (i.e. ~150 BC—AD 1500; Table 1). Modern leporids came from the U.S. Geological Survey and were collected by hunting. They serve as a reference sample for forested landscapes of central Mexico. Approval for analysis of archaeological specimens was granted by the National Institution of Anthropology and History (INAH) in Mexico (Project #: 401.B(4)19.2013/36/1679), and the exportation and destruction of samples was approved by directors of individual site locations. Destructive analysis of modern specimens was approved by Smithsonian’s National Museum of Natural History (Trn # 2059414). Remaining bone material not destroyed during stable isotope analysis is currently stored in the Paleodiet Laboratory at the University of California, San Diego. Specimen numbers and provenience information for each bone sample are listed in S1 & S2 Tables. Here we present a description of each sampled site location in rough chronological order.

**Table 1:** Chronology of Teotihuacan with associated ceramic phases and site contexts.

Period	Calendar Dates	Ceramic Phase	Representative Site Contexts		
Postclassic	AD 1150–1500	Aztec	Túneles y Cuevas		
	AD 900–1150	Mazapan	Puerta 5		
Epiclassic	AD 600–900	Coyotlatelco			
Classic	AD 550–600	Metepec			
	AD 350–550	Xolalpan		Teopancazco	Oztoyahualco
	AD 275–350	Tlamimilolpa late	Moon Pyramid		
	AD 200–275	Tlamimilolpa early			
	AD 150–200	Miccaotli			
Formative	AD 1–150	Tzacualli			
	150–1 BC	Patlachique			

#### Moon Pyramid (150 BC- AD 450)

Moon Pyramid, one of three monumental complexes at Teotihuacan, was first constructed at about AD 100 but was expanded and modified several times until sometime in the 5^th^ century AD [63]. Leporid bones were recovered from within the fill of six superimposed structures. Fill materials likely predate the construction of each phase. Ceramics from associated contexts predominantly date to the Tzacualli phase (1 BCE- 150 AD) but span the Patlachique to early Tlamimilolpa periods (See Fig 1). This estimated temporal range is confirmed by radiocarbon dating of discrete burial events within the pyramid [63]. Because the final building episode occurred around AD 450, older structures and their associated fill predate this time, and most leporid remains likely date to the earliest years of Teotihuacan (BC 1 to 150 AD). Primary excavations of this monumental structure were conducted by Saburo Sugiyama and Ruben Cabrera Castro from 1998–2004 as part of the *Proyecto Pirámide de la Luna*. In total, 56 leporid bones were sampled from the pyramid.

#### Teopancazco (AD 200–550)

Teopancazco was a multiethnic neighborhood center located in the southeastern periphery of the urban core of Teotihuacan. In addition to habitation, the complex served as a center for diverse activities, including craft production, ritual ceremonies, and administration [64]. Teopancazco was occupied between approximately AD 200–650 [65, 66], but the contexts sampled in this study date mainly to the Xolalpan period (350–550 AD). Materials were recovered from excavations directed by Linda R. Manzanilla between 1997 and 2005 as part of the project, *Teotihuacan Elite y Gobierno*. Twenty leporid bones were sampled for this study.

#### Oztoyahualco 15B (AD 350–550)

The Oztoyahualco apartment compound is located northwest of the Pyramid of the Moon in one of the oldest sectors of the city [67], but excavated contexts at the compound (15B:N6W3) date exclusively to the Xolalpan period [16]. Archaeological evidence mentioned above suggests that residents of the compound may have specialized in leporid management or breeding [17]. Excavations between 1985–1988 as part of the project *Antigua ciudad de Teotihuacan*, directed by Linda R. Manzanilla, recovered leporid remains from multiple contexts, and 17 were selected for the present study.

#### Puerta 5 (AD 600–1150)

The Puerta 5 site is a post-Teotihuacan human-made cave/tunnel complex that likely had a ceremonial function. The site is located near the Gate-5 entrance to the archaeological zone, immediately to the east of the Sun Pyramid. Leporid samples primarily come from the two contexts of Cueva III and Cala II and were associated with Coyotlatelco (AD 600–900) and Mazapan (AD 900–1150) phase ceramics. These periods represent the time following the political collapse of Teotihuacan [68]. Puerta 5 was excavated under the direction of Eduardo Matos Moctezuma and Ruben Cabrera as part of the salvage excavation, *Proyecto Especial Teotihuacán 1992–1994* [69]. Twenty-nine bones were sampled for this study.

#### Túneles y Cuevas (AD 1150–1500)

Leporids from two tunnels with Epiclassic and late-occupation Postclassic period contents were collected at Cueva Pirul and Cueva de las Varillas, hereafter jointly referred to as *Cuevas*. These tunnels are adjacent to each other and are located east of main axis of the city, immediately behind the Pyramid of the Sun. Although the tunnels were in use as early as the Coyotlatelco phase, most selected leporids are associated with Aztec phase (AD 1150–1500) materials [70]. These human-made tunnels were used for a variety of purposes, including habitation, storage, ritual, burial, and extraction of construction material [71]. Samples were recovered during excavations of the *Tunnels and Caves at Teotihuacan* project, directed by Linda R. Manzanilla in 1992–1996 [72]. Twelve bones from fill contexts were sampled for analysis.

#### Modern (AD 1892–1966)

**Fig 3:**
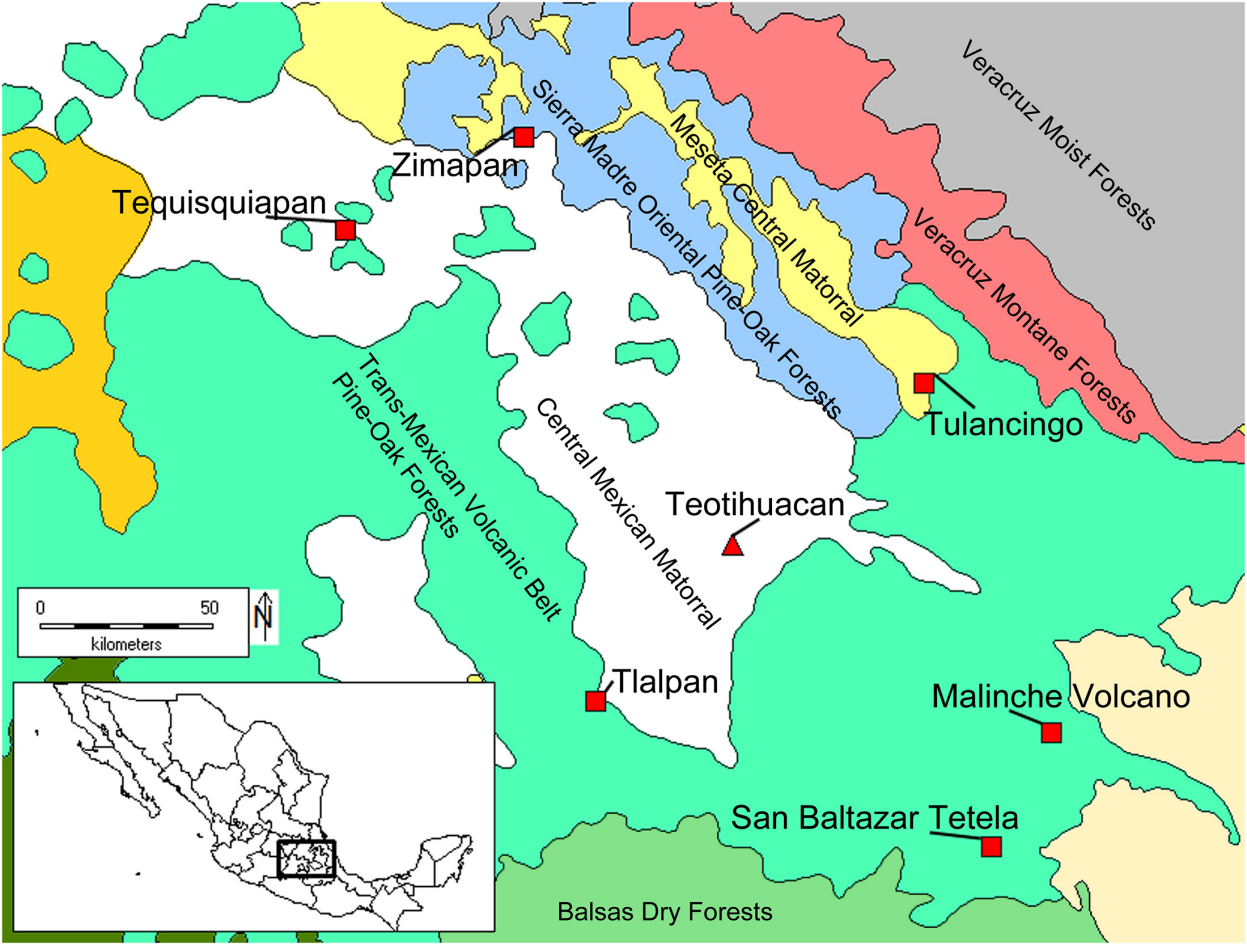
Map displaying the location of Teotihuacan (red triangle) in relation to the location of modern leporid specimens (red squares) and nearby terrestrial ecoregions (shaded regions). Terrestrial ecoregion data were obtained from the World Wildlife Federation’s online spatial dataset (http://www.worldwildlife.org/publications/terrestrial-ecoregions-of-the-world). The map was generated using DIVA-GIS [76].

To provide a comparative sample, 13 modern leporids were sampled from the Smithsonian Institution’s mammalian vertebrate collection. These wild specimens provide a reference sample for leporids from known environmental ecoregions near the Basin of Mexico. Unfortunately, no specimens were available from the Teotihuacan valley itself. Museum specimens were originally obtained through hunting in the regions of Tlalpan of Distrito Federal, Tulancingo and Zimapan of Hidalgo, Tequisquiapan from Queretaro, and from San Baltazar Tetla and Malinche Volcano in Puebla. Areas represent Central Mexican matorral deserts and pine-oak forest landscapes, similar to the general setting of Teotihuacan but with more of an emphasis on forested habitats (S1 Table). Fig 3 depicts the location of modern leporid source locations relative to Teotihuacan. To account for the decrease in atmospheric ^13^CO_2_ because of the industrial (Suess) effect [73], the 20^th^ century (1966) leporid *δ*^13^C_apatite_ values are corrected by +0.69‰ to match archaeological values, assuming a preindustrial atmospheric δ^13^C value of -6.35‰ [74] and an approximated 1966 value of -7.04‰ [75]. The 19^th^ century samples (1892, 1893, 1896) are assumed to have lived under an atmospheric δ^13^C value of approximately -6.60‰ [75], and leporid *δ*^13^C_apatite_ from these years are corrected by +0.25‰.

### Laboratory Procedures

Initial cleaning of bone specimens was accomplished by ablating the bone surface with a Dremel engraving tool to remove dirt and other surface adherents. Spongy trabecular bone was also removed through this process. All samples then received a sonic bath (10 minutes) in approximately 50 mL of acetone followed by two consecutive sonic baths in approximately 50 mL of double-distilled water (5 minutes each). Preparation of bone bioapatite followed procedures similar to those of Koch et al. (1997). Powdered bone was extracted along the proximal-distal axis of long-bones with a diamond-tipped engraving bit, or was achieved by reducing bone fragments to powder in an agate mortar and pestle. Powdered samples were then passed through a micromesh (200 opening, 75 μm) sieve to ensure consistency of sample size. Samples were then treated for 24 hours with 2% bleach (NaOCl) and 24 hours with 0.1M acetic acid (CH_3_COOH), and rinsed three times with doubled distilled and deionized water between treatments. Powdered and cleaned samples were dried for 24 hours at 60°C in a laboratory oven. Stable isotope analyses were conducted at the Scripps Institute for Oceanography’s Analytical Facility under the direction of Dr. Bruce Deck. CO_2_ gas was liberated from bone carbonate with pure phosphoric acid (H_3_PO_4_) at 70°C and sampled from within glass vials by an autosampler. Isotope ratios of carbon and oxygen were measured with Gas Bench II coupled to a Thermo-Finnigan Delta Plus XP mass spectrometer. Stable carbon isotope ratios were calibrated to the Vienna Pee Dee Belemnite (VPDB) international standard, and stable oxygen isotope ratios were presented relative to the Vienna Standard Mean Ocean Water (VSMOW) by using standards calibrated against NBS-18 and NBS-19. A constant CO_2_ in He standard (GS) was run to determine drift with time, and two fine powdered CaCO_3_ working standards (MKS), with varying amounts for peak intensity correction were run after every eight leporid samples. Over the period of data analysis, accuracy and precision were assessed by analyzing an additional laboratory CaCO_3_ standard after every 8 samples, resulting in means of *δ*^13^C = -12.48 ±0.06‰ (N = 20, 1 SD) and *δ*^18^O = 11.87 ± 0.12‰ (N = 20, 1 SD), which compared well to expected values of *δ*^13^C = -12.5 and *δ*^18^O = 11.9‰.

To assess the degree of bone mineral diagenesis, a random sample (N = 101, 74%) of archaeological specimens was analyzed by Fourier-Transform Infrared spectroscopy with the Attenuated Total Reflection technique (FTIR-ATR) at the Department of Chemistry and Biochemistry at the University of California at San Diego. FTIR-ATR analysis was used to obtain the infrared splitting factor (IR-SF) and carbonate to phosphate (C/P) ratios, which can be considered proxies for the presence of post-depositional alteration to the mineral structure of bone [77–79]. Several milligrams of finely powdered bone, which had already received pre-treatment as described above, were analyzed on a Smart-iTR diamond crystal ATR stage equipped to a Thermo Scientific Nicolet 6700 FT-IR spectrometer. Spectra were collected in 100 scans and controlled for background variance. Following calibration experiments on modern bone in the Paleodiet Laboratory and in consultation with previous literature [79, 80], we anticipated that bone samples containing biogenic values would exhibit carbonate to phosphate (C/P) ratios between approximately 0.10–0.50 and infrared splitting factor (IR-SF) values between 2.0–4.0.

### Statistical Analyses

Parametric tests were used to identify differences between groups of two (independent samples t-test) and more than two (between subjects analysis of variance [ANOVA]). However, to compare differences in stable isotope values between the Oztoyahualco site context and the pooled sample of values from other site locations (Moon Pyramid, Teopancazco, Puerta 5, & Cuevas), we used the non-parametric Mann-Whitney U test, due to the very different sample sizes. To assess whether stable carbon and oxygen isotope values covary, a bivariate Pearson’s correlation was used. Significance was assumed at α = 0.05. All analyses were conducted in Statistical Package for the Social Sciences (SPSS v.22).

### Two-source mixing model

To calculate the approximate percentage of C4 and/or CAM plants in the diet of analyzed leporids, we used a simple two-source mixing model ([81] pp 139–142). Bone *δ*^13^C_apatite_ was converted to reflect the proportion of C4/CAM plants in the diet (%C4CAM) through the following equation:

$$\%\text{C}4\text{CAM}=(\delta_{\text{sample}}–\delta_{\text{C}3})/(\delta_{\text{C}3}-{\;\delta }_{\text{C}4\text{CAM}}),$$

where δ_sample_ represents the measured bone stable carbon isotope ratio minus the diet-apatite offset of 9.7‰ [29], δ_C3_ represents the assumed *δ*^13^C end-member value of C3 plants, and *δ*_C4CAM_ represents the assumed end-member value for C4 and CAM plants. End-member δ^13^C values were estimated by using values from highland Mexico plants acquired from modern markets and from wild samples from the Mitla and Yagul cave region of Oaxaca by Warinner et al. [25]. To avoid potential confounds of diagenesis, archaeological plant samples were not included in calculating the end-member averages. Their results reflect a C3 average of -27.6 ±2.2‰ (n = 221, 1SD) and a combined average of C4 and CAM plants of -13.5 ± 2.3 (n = 221, 1SD). We added +1.5‰ to their modern plant values to adjust for the industrial effect, resulting in an adjusted C3 mean of -26.1‰ and an adjusted C4/CAM mean of -12.0‰. Hence, the following equation:

$$\%\text{C}4\text{CAM}=((\delta^{13}{\text{C}}_{\text{apatite}}–\;9.7)-\left(-26.1\right))/14.1$$

Results from the mixing model, however, are to be considered rough estimates due to uncertainties in the offset between leporid bone δ^13^C_apatite_ and dietary δ^13^C, analytical error inherent in sample processing and analysis, variation in local plant values, and uncertainties concerning actual plant δ^13^C values in the Teotihuacan valley. Our use of the model was thus intended as a means of approximating relative contributions of C3 and C4/CAM plants to leporid diets from different contexts, and should not be seen as a precise measure of actual dietary input. In fact, because maize tends to exhibit δ^13^C values up to two per mil higher than other C4 and CAM plants [25], our calculations might overestimate %C4CAM in leporid diet if maize was the primary plant consumed.

## Results

Stable isotope results for each modern leporid specimen are reported in S1 Table and results for archaeological specimens are reported in S2 Table. Assessments of diagenesis found several specimens that fell outside of predetermined acceptable parameters. Five apatite samples with C/P ratios <0.10, three apatite samples with IR-SF values < 2.00, and five apatite samples with IR-SF values >4.00 were excluded from subsequent discussions (See S2 Table). One *δ*^18^O_apatite_ value (11.9‰; sample AS-0335) was an extreme outlier and was excluded because the low value was likely due to an analytical problem and/or contamination of the sample. Across all archaeological contexts and time periods the means were: *δ*^13^C_apatite_ = -7.8 ± 2.4‰ (N = 114, 1 S.D.) and *δ*^18^O_apatite_ = 26.0‰ (N = 113, 1 S.D.). The modern reference specimens exhibited mean values of *δ*^13^C_apatite_ = -12.4 ± 1.8‰ (N = 13, 1 S.D.) and *δ*^18^O_apatite_ = 26.1‰ ± 1.4 (N = 13, 1 S.D.).

Independent samples t-tests were conducted to compare mean stable isotope ratios of the two genera (*Lepus* and *Sylvilagus*) across the archaeological bone sample as a whole. No significant difference between the genera were found in *δ*^13^C_apatite_ values (t[110] = -0.806, *p* = 0.422) or *δ*^18^O_apatite_ values (t[110] = 0.428, *p* = 0.669). We also evaluated stable isotope ratios between the genera and between individual site locations using a two-way ANOVA (factorial ANOVA with two factors), testing for the main effects of genus and context, and for the genus*context interaction. This permitted us to compare differences between the genera both within and across site contexts. For *δ*^13^C_apatite_, the main effect for genus was not significant (F[1,102] = 3.361), *p* = 0.070), but the main effect of site context was significant (F[4,102] = 5.365, *p* = 0.001). Importantly, the interaction between genus and site was not significant for *δ*^13^C_apatite_ (F[4,102] = 0.590, *p*<0.670) or for *δ*^18^O_apatite_ (F[4,102] = 0.834, *p*<0.507). However, *Lepus* and *Sylvilagus* values are notably different from each other at the Cuevas site, but the small sample size from this context makes any interpretations regarding this observation speculative. Subsequent contextual comparisons thus combine *Sylvilagus* and *Lepus* specimens. Summary statistics for each site context are found in Table 2.

**Table 2:** Descriptive statistics for stable carbon and oxygen isotope values grouped by genus and selected site locations.

		*δ*^13^C apatite-VPDB (‰)			*δ*^18^O apatite-VSMOW (‰)		
		*n*	Mean	S.D.	*n*	Mean	S.D.
Moon Pyramid	*Sylvilagus*	35	-8.8	2.1	34	26.7	1.9
	*Lepus*	16	-8.4	3.1	16	25.1	2.1
	Subtotal	51	-8.7	2.5	50	26.2	2.1
Teopancazco	*Sylvilagus*	14	-7.1	1.1	14	27.5	1.6
	*Lepus*	5	-6.8	2.2	5	26.7	1.0
	Subtotal	19	-7.0	1.4	19	27.3	1.5
Oztoyahualco	*Sylvilagus*	11	-6.2	2.3	11	24.9	2.4
	*Lepus*	4	-5.1	1.2	4	26.4	2.2
	Subtotal	15	-5.9	2.1	15	25.3	2.4
Puerta 5	*Sylvilagus*	17	-7.4	2.1	17	24.3	1.4
	*Lepus*	2	-6.2	.8	2	24.8	1.8
	Subtotal	19	-7.3	2.1	19	24.4	1.4
Cuevas	*Sylvilagus*	6	-9.9	1.8	6	26.0	3.3
	*Lepus*	4	-7.5	1.4	4	26.2	2.2
	Subtotal	10	-8.9	2.0	10	26.1	2.8
Modern	*Sylvilagus*	12	-12.5	1.6	12	26.0	1.4
	*Lepus*	1	-10.5	.	1	27.5	.
	Subtotal	13	-12.4	1.6	13	26.1	1.4
Total	*Sylvilagus*	95	-8.5	2.6	94	26.1	2.1
	*Lepus*	32	-7.5	2.7	32	25.7	2.0
	Subtotal	127	-8.3	2.7	126	26.0	2.1

**Fig 4:**
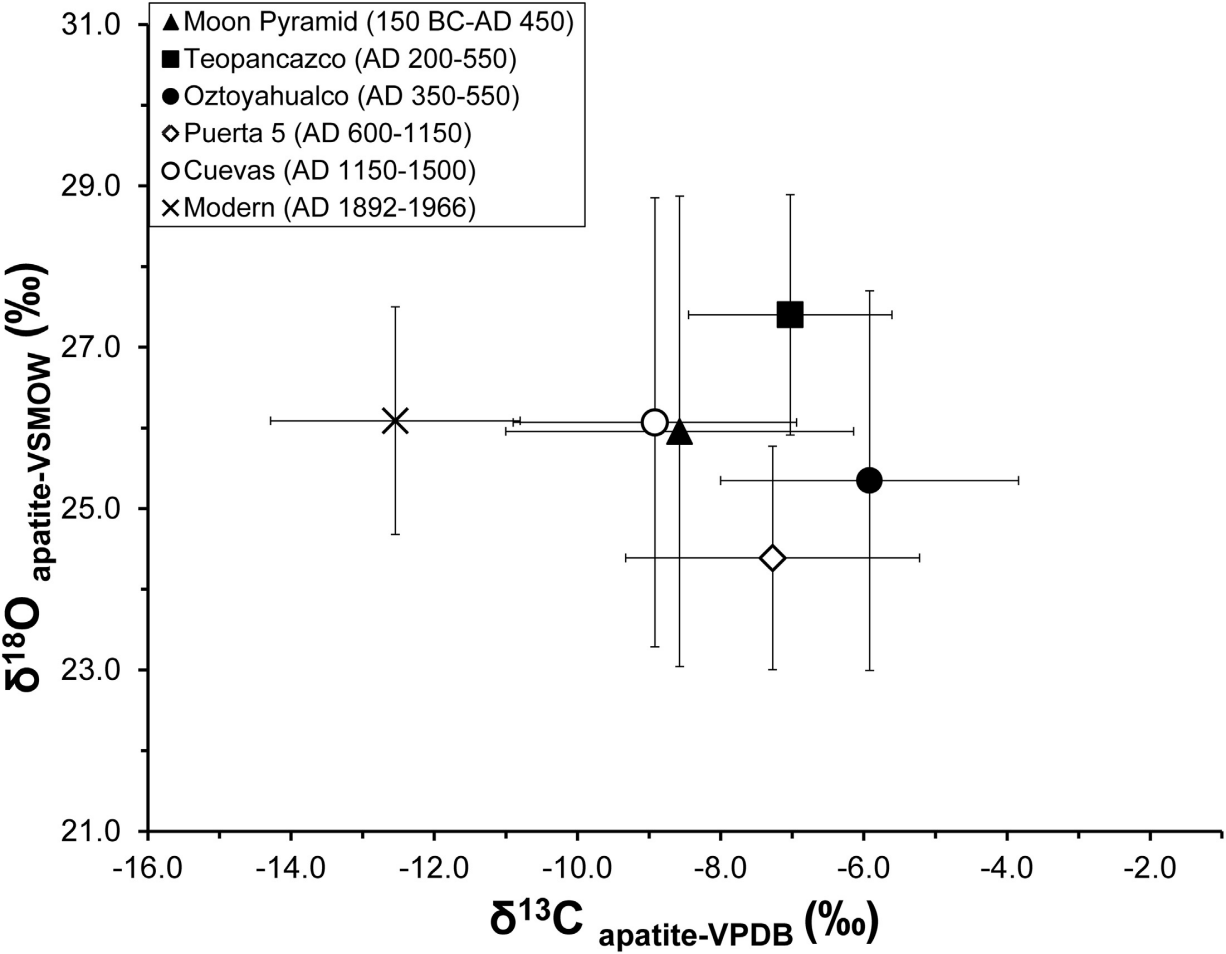
Scatterplot of stable carbon and oxygen stable isotope values grouped by site locations. Markers of the proposed leporid breeding complex of Oztoyahualco are highlighted as filled black circles.

**Fig 5:**
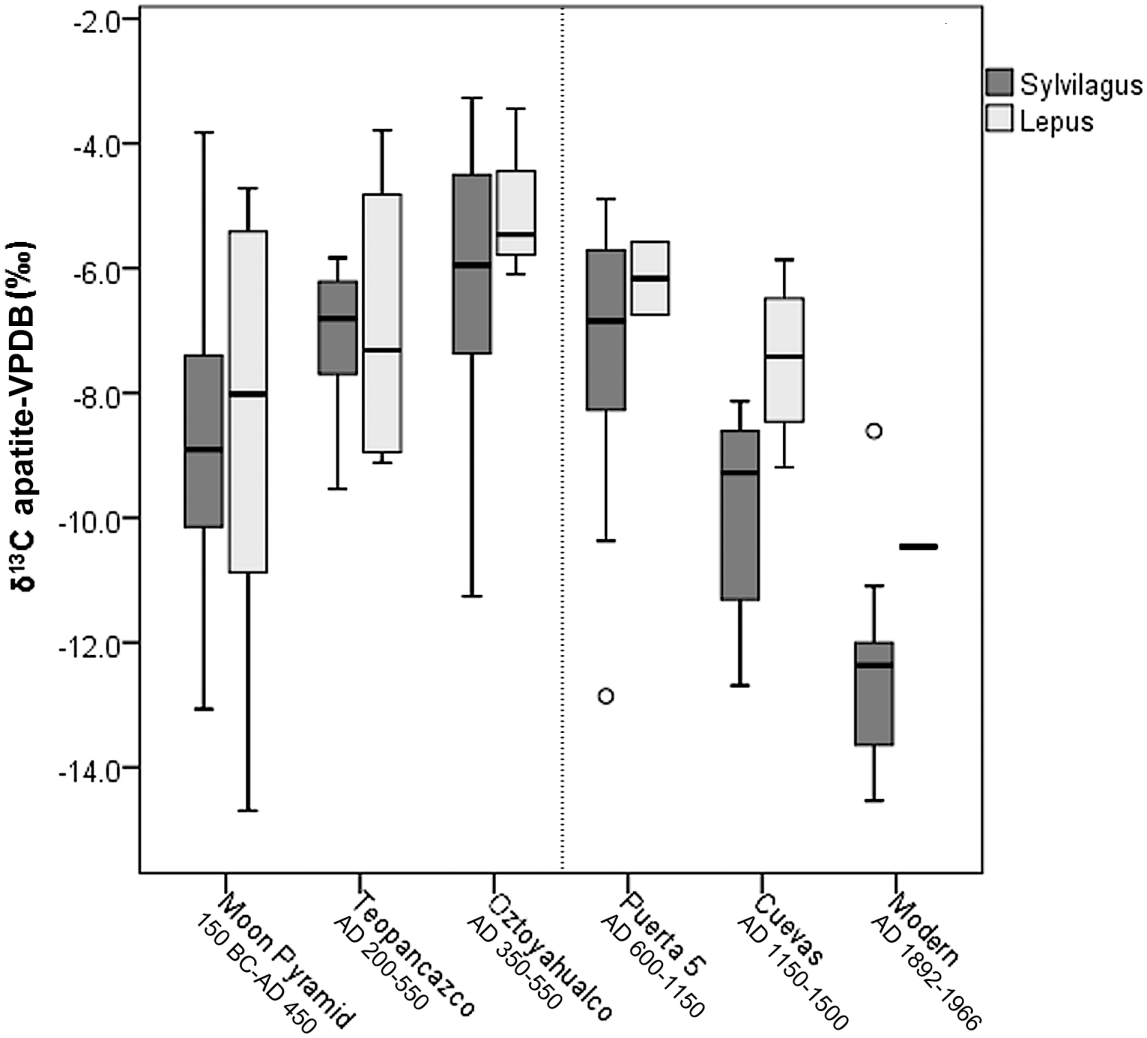
Box plots depicting distributions of stable carbon isotope values of leporid bone apatite from each site location. Data are separated by genus and are arranged in rough chronological order moving from left (oldest) to right (most recent). The dashed line represents the approximate date of the sociopolitical collapse of Teotihuacan.

**Fig 6:**
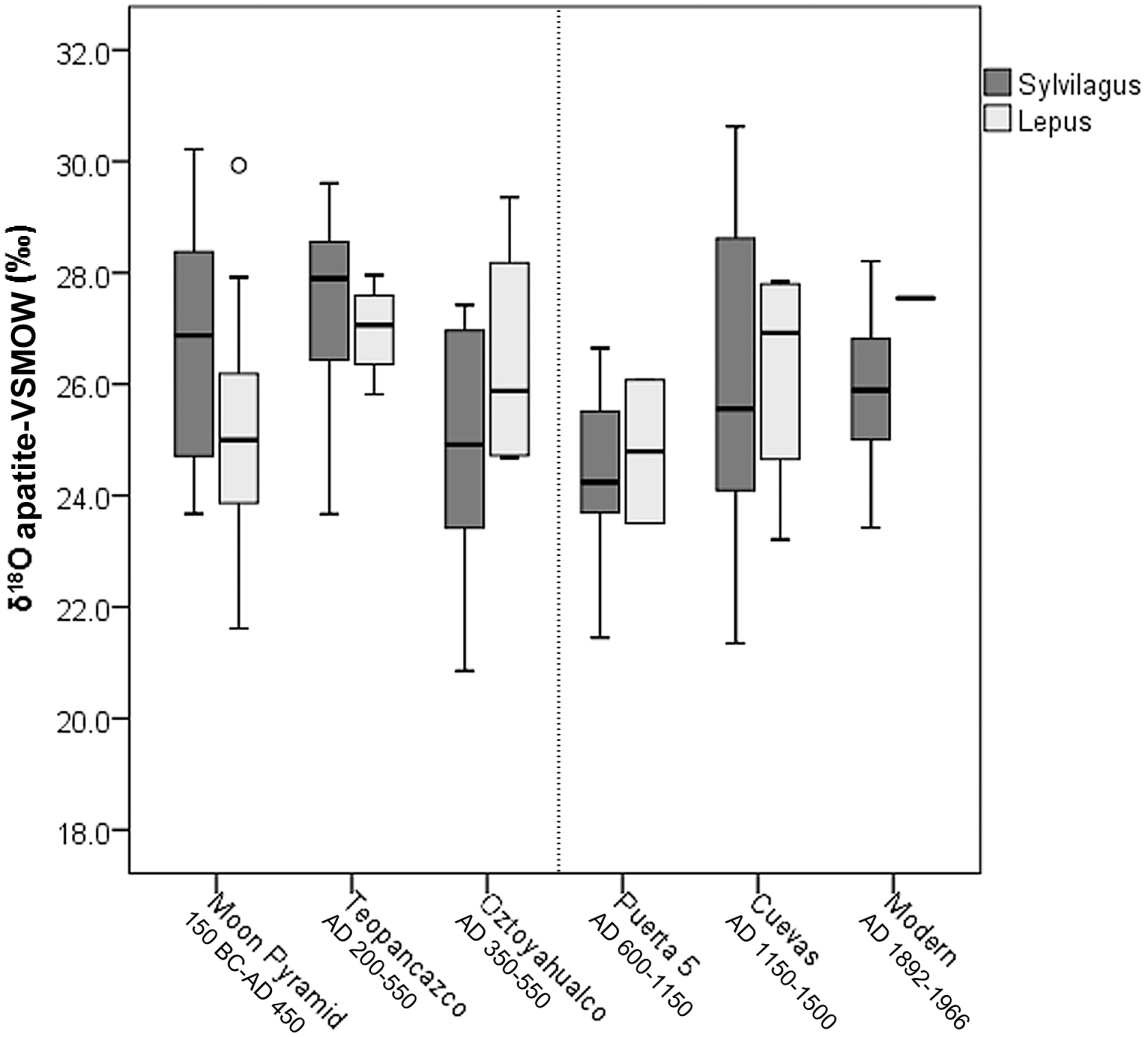
Box plots depicting distributions of stable oxygen isotope values of bone apatite from each site location. Data are separated by genus and are arranged in rough chronological order moving from left (oldest) to right (most recent). The dashed line represents the approximate date of the sociopolitical collapse of Teotihuacan.

To investigate differences in leporid stable isotope values across site contexts within the archaeological sample, we conducted a one-way between subjects ANOVA. Significant differences were found in *δ*^13^C_apatite_ (F[4, 107] = 5.8, *p*<0.001). Post-hoc Tukey tests indicate that Oztoyahualco leporids had significantly higher *δ*^13^C_apatite_ values than those of the Moon Pyramid and the Cuevas. They also had higher mean *δ*^13^C_apatite_ than every other context (see Fig 4 and Table 2). *δ*^13^C_apatite_ values, then, demonstrate that leporids from Oztoyahualco consumed a diet much higher in C4/CAM foods than those from the modern reference sample and indeed from every other site location within the city (Fig 5). Significant differences were also found in *δ*^18^O_apatite_ means between site contexts (F[5, 120] = 4.704, *p*<0.001), but no obvious temporal trends were observed (Fig 6). Post-hoc Tukey tests revealed significant differences in *δ*^18^O_apatite_ between Oztoyahualco and Teopancazco (*p* = 0.046), between Puerta 5 and Teopancazco (*p*<0.001), and between Puerta 5 and the Moon Pyramid (*p* = 0.008). To test the hypothesis that Oztoyahualco leporids would exhibit higher *δ*^13^C_apatite_ than the combined Teotihuacan sample (Moon Pyramid, Teopancazco, Puerta 5, & Cuevas), we used a Mann-Whitney U test to compare their distribution. Significant differences were found for *δ*^13^C_apatite_ (*U* = 329, *p* = 0.001) but not *δ*^18^O_apatite_ (*U* = 636, *p* = 0.434).

## Discussion

### Temporal and Spatial Considerations

Carbon stable isotope ratios from bone apatite in archaeological specimens were significantly higher than the modern specimens. Combining genera, a two-source mixing model of bone apatite stable carbon isotope mean values found that Teotihuacan leporids consumed a diet of roughly 62% C4/CAM plants while leporids from modern reference sample consumed a diet of approximately 29% C4/CAM plants. Such differences were anticipated as the modern reference sample included leporids from more forested ecoregions that likely had fewer locally available C4 and CAM plants. Mean *δ*^18^O_apatite_ values of ancient and modern specimens, however, were essentially identical (26.0‰ vs 26.1‰), suggesting that relative humidity in the Teotihuacan valley during the pre-Hispanic era was broadly similar to that of the modern reference.

To explore changes in leporid ecology through time, isotopic results were assessed according to the approximate chronological order of sampled site locations (Figs 5 and 6). Although all five locations represented different temporal ranges, significant overlap existed between chronologies at each site (See Table 1). Moon Pyramid contexts (150 BC—AD 450) overlapped in time with both the early occupations of Teopancazco (AD 200–550) and Oztoyahualco (AD 350–550), which themselves overlapped in time. Additionally, although leporids from the Cuevas contexts date mostly to the Aztec period (AD 1150–1500) by ceramic association, several leporids came from levels that contained some Coyotlatelco period (AD 600–900) ceramics and thus may overlap slightly in time with Puerta 5 specimens.

Nevertheless, when arranged in this rough chronological order, some notable trends were observed. Stable carbon isotope values appear to increase through the Formative and Classic periods, achieving an apex at Oztoyahualco, and then decreasing after the political and demographic collapse of the city through the Epiclassic and Postclassic occupations at Puerta 5 and the Cuevas (Fig 5). This pattern was apparent in both *Sylvilagus* and *Lepus* specimens. Indeed, the earliest temporal context, the Moon Pyramid (150 BC– 450 AD), exhibited the second lowest mean *δ*^13^C_apatite_ value of the sample. Oztoyahualco leporids, deposited during the peak of socio-political complexity of the city (AD 350–550), exhibited the highest mean *δ*^13^C_apatite_. And the final occupation of Teotihuacan at the Cuevas context (AD 1150–1500) contained leporids with the lowest *δ*^13^C_apatite_ mean of the archaeological sample.

Three possible and interacting factors may account for the observed increase in leporid *δ*^13^C_apatite_ during the Classic period occupation. First, as the human population of the Teotihuacan valley grew and as agricultural landscapes expanded proportionately, leporids became increasingly integrated into the human ecological system, evidenced by their high consumption of C4/CAM plants such as maize, nopal, maguey, and amaranth. As both *Sylvilagus* and *Lepus* are strongly attracted to agricultural fields, crop raiding may have been a significant source for the high C4/CAM signal. Secondly, human clearing of nearby pine and oak forests may have opened new habitats for C4 grasses at the expense of C3 plants, potentially serving as an additional influence on the trend of increasing bone *δ*^13^C_apatite_ values through the Classic Period. It is notable that leporids from the earliest and latest occupations of the valley, which were also the periods associated with the lowest human populations, exhibited *δ*^13^C_apatite_ values closest to the modern reference sample, which consisted of leporids from mostly pine-oak forest ecoregions (Fig 4). Low values from the early Moon Pyramid occupation (150 BC—AD 450) suggest that prior to the period of major growth of the city, the local landscape was characterized as having greater forest cover and less C4/CAM vegetation. These findings are in agreement with research on paleosol profiles by McClung de Tapia and colleagues [36], who found that soils from the Teotihuacan era were characterized by deforestation, burning, compaction, and erosion in comparison to earlier periods. Low *δ*^13^C_apatite_ values of leporids during the final phase of the city at the Cuevas context, suggest that forests around the valley may have been regenerating at this time. Finally, the practices of domestic management and direct provisioning of leporids with human-cultivated C4 plants, discussed more below, may have occurred alongside the previous two factors and may have been a significant source of high *δ*^13^C_apatite_ at Teopancazco and Oztoyahualco.

Stable oxygen isotope values do not follow a similar pattern as the stable carbon isotope values over time. A Pearson correlation found that the relationship between these variables was not significant (*R* = -0.003, *p* = 0.971). If *δ*^13^C_apatite_ and *δ*^18^O_apatite_ showed similar changes through time, the patterning would have supported the notion that climatic changes were responsible for the rising and falling prevalence of C4/CAM plants, which prefer warm, dry, and sunny environments. The fact that leporid *δ*^13^C_apatite_ does not correlate with *δ*^18^O_apatite_ implies that non-climatic factors, such as landscape modification or direct provisioning, had more influence over C4/CAM plant abundance in leporid diet than did climatic variability.

Some variation in *δ*^18^O_apatite_ values, however, was observed between Teotihuacan site contexts, with Teopancazco (AD 200–550) exhibiting relatively high values and Puerta 5 (AD 600–1150) exhibiting relatively low values. One possible explanation for the observed *δ*^18^O_apatite_ differences may be differential sources of acquisition for leporids within the Teotihuacan Valley or beyond. Oxygen isotope variation may reflect different hunting locations, particularly garden hunting, as plant water stable oxygen isotope ratios can vary due to different sources of irrigation water [82]. Leporids acquired from different fields within the valley or from other locations across the broader region could reflect differences in source water or microclimates. Additionally, short-term climatic variation could have resulted in the observed differences in *δ*^18^O_apatite_ between site contexts, with Teopancazco (mean = 27.3 ± 1.5‰ [n = 14, 1SD]) representing the period with the lowest relative humidity and Puerta 5 (mean = 24.4 ± 1.4‰ [n = 19, 1SD]) representing the period with the highest. Intra-site variation in *δ*^18^O_apatite_ is also somewhat consistent with the paleosol findings of McClung de Tapia et al [76], which identified variability in plant phytoliths and the presence of carbonates across soil layers, both suggesting a degree of environmental variability in temperature and humidity over the occupational history of the city.

### Leporid Management at Oztoyahualco

Our hypothesis that leporids from Oztoyahualco would exhibit higher *δ*^13^C_apatite_ values than leporids from other site locations was supported. The mean *δ*^13^C_apatite_ value of -5.9‰ at Oztoyahualco corresponded to a total diet of roughly 74% C4/CAM plants, and was significantly higher (*p* = 0.001) than the combined mean for other archaeological site locations (-8.3‰). The exceptionally high stable carbon isotope values from Oztoyahualco provide strong support to the notion that this residential complex specialized in the production of leporids. High *δ*^13^C_apatite_ values, in combination with the archaeological and iconographic evidence for leporid management, suggest that human-leporid interactions qualitatively changed during the Xolalpan period at this complex. What was once a hunter-prey relationship, centered on the dynamics of leporid crop raiding and human garden hunting, may have transformed into one of active management and controlled reproduction with humans directly provisioning cottontails and jackrabbits C4 and CAM agricultural products. The amount for C4/CAM plant consumption by Oztoyahualco leporids is less than some other animals raised under captivity in the New World, such as tropical birds from Paquimé in Chihuahua, which may have consumed diets of up to 100% C4/CAM products [33], but higher than some domesticated dogs in the Maya region [34]. Nevertheless, the diet of Oztoyahualco leporids was significantly higher in C4/CAM plants than the average for other Teotihuacan contexts.

Together, the archaeological evidence for leporid management and the isotopic data are consistent with the notion that leporid production was a specialized activity at Oztoyahualco, but they do not necessitate that all leporid raising or provisioning occurred on site. Social affiliates or economic partners of the compound may have kept leporids in off-site managed landscapes near agricultural fields in a manner similar to medieval European rabbit management practices [83]. Farmers could have intentionally dedicated particular garden patches for leporid consumption, trapping individuals as needed. Indeed, similar practices have been described for modern Maya farmers of Mexico who dedicated managed forest patches to attract animals for hunting ([84] pp 15–19). It is possible that leporids not raised at the residential compound were brought to Oztoyahualco as a center for butchering and processing carcasses for meat and secondary products. In fact, several specimens exhibiting exceptionally low stable carbon isotope values at Oztoyahualco may identify leporids originating from wild populations beyond the immediate Teotihuacan valley, suggesting that hunting occurred alongside active management.

Because both *Sylvilagus* and *Lepus* exhibited higher *δ*^13^C_apatite_ values in comparison to other site locations, residents of Oztoyahualco may have practiced a generalized form of animal management. Instead of concentrating on the mass production of single species, as do modern husbandry industries, leporid specialists of Teotihuacan may have managed diverse mixed-genus populations. Indeed, six different leporid species were discovered within the complex [17]. At least some scale of domestic breeding on site, however, is suggested by the archaeological evidence discussed above. Regardless of the manner by which leporids were acquired, these animals appear to have been particularly important to Oztoyahualco’s residents, as indicated by the presence of the unique stone sculpture of a rabbit found within a public courtyard. Manzanilla [15] has suggested that this sculpture represents the totem animal for one of the households of the residential complex. Another possible and complementary interpretation is that the sculpture signified the primary economic specialization of the compound’s residents.

## Conclusion

Stable isotope data produced in this study inform our understanding of the long-term human-leporid relationships at the pre-Hispanic New World city of Teotihuacan. Carbon isotope data in particular provide strong evidence of Teotihuacan’s growing impact on the local environment through the Classic period. Higher *δ*^13^C_apatite_ values of Teotihuacan-era leporids in comparison to a modern reference sample suggest that these animals acquired a significant portion of their diet from C4 and CAM agricultural products, likely resulting from a combination of consuming local C4/CAM plants, the raiding of agricultural fields, and through the practices of human provisioning and management.

Based on archaeological evidence for butchering, the high frequency of leporid remains, and the presence of a unique rabbit sculpture, we hypothesized that leporids from the Oztoyahualco complex would display greater evidence C4/CAM plant consumption due to their direct provisioning by humans. The finding that the mean leporid *δ*^13^C_apatite_ value from Oztoyahualco was the highest of the sample set, indicating high consumption of plants such as maize, maguey, nopal, and amaranth, is consistent with this hypothesis and provides strong support for the specialized procurement of leporids. Small mammal management within the city could have served as a means to channel surplus C4/CAM agricultural products to provision cottontails and jackrabbits, effectively converting excess carbohydrates into high quality protein and economically valuable secondary products, such as fur, hide, glue, and bones for tools.

Although no large-bodied terrestrial herbivores were available for domestication in Mesoamerica, inhabitants of pre-Hispanic Mexico engaged in relationships with many smaller and a more diverse non-domesticated fauna [85, 86], such as cottontails and jackrabbits, but also turkeys (*Meleagris gallopavo*) [87], turtles (*Dermatemys mawii*) [88], and insects such as cochineal (*Dactylopius coccus*) and bees (*Melipona beecheii*) [89, 90]. The chronological sample from Teotihuacan, moreover, suggests that such relationships may have become more intensive through time, perhaps in response to dwindling populations of large mammals such as deer (*Odocoileus virginianus*). Although human-animal relationships were not as archaeologically visible or as impactful to local environments as human-ungulate relationships in Eurasia, human-animal interactions were nevertheless important components of the emergence and organization of complex societies of ancient North America, supplying sources of high-quality protein and other economically valuable products to support dense urban populations.

## Supporting Information

S1 Table Stable isotope data and contextual information for modern leporid specimens. Stable carbon isotope values have been corrected for the industrial (Suess) effect as detailed in the text.
(XLSX)

S2 Table Provenience information, stable isotope results, and FTIR-ATR data for each archaeological specimen in the study. Data points highlighted in grey were excluded from analyses due to possible diagenesis or analytical error.
(XLSX)
